# Number and timing of antenatal HIV testing: Evidence from a community-based study in Northern Vietnam

**DOI:** 10.1186/1471-2458-11-183

**Published:** 2011-03-25

**Authors:** Nguyễn TT Hạnh, Tine M Gammeltoft, Vibeke Rasch

**Affiliations:** 1 Department of Population, Institute for Preventive Medicine and Public Health, Hanoi Medical University, No.1 Ton That Tung Street, Khuong Thuong, Dong Da, Hanoi, Vietnam; 2 Department of Anthropology, University of Copenhagen, Øster Farimagsgade 5, DK-1353 Copenhagen K, Denmark; 3 Department of International Health, Immunology and Microbiology, Faculty of Health Sciences, University of Copenhagen, Øster Farimagsgade 5, DK-1014 Copenhagen, Denmark; 4 Department of Obstetrics and Gynaecology, Odense University Hospital, 5000 Odense C, Denmark

## Abstract

**Background:**

HIV testing for pregnant women is an important component for the success of prevention of mother-to-child transmission of HIV (PMTCT). A lack of antenatal HIV testing results in loss of benefits for HIV-infected mothers and their children. However, the provision of unnecessary repeat tests at a very late stage of pregnancy will reduce the beneficial effects of PMTCT and impose unnecessary costs for the individual woman as well as the health system. This study aims to assess the number and timing of antenatal HIV testing in a low-income setting where PMTCT programmes have been scaled up to reach first level health facilities.

**Methods:**

A cross-sectional community-based study was conducted among 1108 recently delivered mothers through face-to-face interviews following a structured questionnaire that focused on socio-economic characteristics, experiences of antenatal care and HIV testing.

**Results:**

The prevalence of women who lacked HIV testing among the study group was 10% while more than half of the women tested had had more than two tests during pregnancy. The following factors were associated with the lack of antenatal HIV test: having two children (aOR 2.1, 95% CI 1.3-3.4), living in a remote rural area (aOR 7.8, 95% CI 3.4-17.8), late antenatal care attendance (aOR 3.6, 95% CI 1.3-10.1) and not being informed about PMTCT at their first antenatal care visits (aOR 7.4, 95% CI 2.6-21.1). Among women who had multiple tests, 80% had the second test after 36 weeks of gestation. Women who had first ANC and first HIV testing at health facilities at primary level were more likely to be tested multiple times (OR 2.9 95% CI 1.9-4.3 and OR = 4.7 95% CI 3.5-6.4), respectively.

**Conclusions:**

Not having an HIV test during pregnancy was associated with poor socio-economic characteristics among the women and with not receiving information about PMTCT at the first ANC visit. Multiple testing during pregnancy prevailed; the second tests were often provided at a late stage of gestation.

## Background

Prevention of mother-to-child transmission of HIV (PMTCT) has considerably reduced the rate of HIV-infection among newborn infants [1,2]. PMTCT services are often integrated with antenatal and obstetric care at different levels of the health care system [3-5]. The rates of HIV infection among pregnant women in Asia are not high, generally 1-2% [6] and the epidemic is mainly concentrated in high risk populations, known to be injecting drug users, sex workers and their clients, and men who have sex with men. However, the epidemic is now dramatically expanding into lower-risk populations through transmission via sexual partners, increasing risks of transmission among women of reproductive age. Among people living with HIV/AIDS in the region, the proportion of females rose from 19% in 2000 to 35% in 2008 [7]. Therefore, in order to protect women from HIV and reduce the rate of HIV transmission among infants born to HIV-infected mothers, PMTCT programmes have been launched in a number of Asian countries, including Thailand, India, Malaysia, China and Vietnam [5,8-10].

HIV counseling and testing (HCT) during pregnancy is an important initial component for successful PMTCT. Pregnant women are advised to be tested as early as possible, preferably during the first trimester [3,11,12]. In spite of efforts made to scale up PMTCT in areas hard hit by the HIV epidemic, a lack of HIV testing during pregnancy has been reported from several studies. In community-based studies in India and Uganda, where PMTCT programmes have been implemented, it has been documented that less than 10% of pregnant women in rural areas were tested during pregnancy [5,13]. The main barriers to HCT included difficult access to antenatal care (ANC), unavailability of HCT services, lack of knowledge on PMTCT, fear of stigma if found to be HIV positive, and lack of awareness about HCT due to limited communication between women and providers during ANC [5,13-15].

According to the revised recommendations of CDC for HIV testing, a repeat test should be provided for HIV-negative pregnant women before 36 gestational weeks, especially in high prevalence settings [1]. This recommendation is founded on the 'window period' of HIV infection, that is, the time between HIV infection and the production of antibodies, which may last 3-6 months. During this period, the test results may be negative even when the person is infected with HIV [16]. To avoid false negative results, HIV tests are recommended to be done three months after potential exposure to infection. Thus, if a woman is tested for HIV before 28 weeks of gestation and the result is negative, a second test is recommended. However, the provision of a second test should be considered in connection with the actual HIV-prevalence and the individual woman's risk or symptoms of HIV-infection [1,11]. Though antenatal HIV-screening generates cost-saving and health benefits in both high and low-income countries [17-19], provision of a repeat test during pregnancy also incurs costs, in terms of time, transport and test payment, as well as consuming human resources and using health facility (HF) infrastructure. Therefore, unnecessary repeat HIV testing during pregnancy both exposes the individual to emotional stress and imposes financial burdens on the health care system [19]. Moreover, if the second test is provided after 36 gestational weeks or at the time of labour, the woman and her unborn child will not gain the full benefits of the PMTCT service offered. The guidelines for antenatal HIV testing should therefore stress that women should only be offered the relevant number of tests for their situation, and at a relevant time in their pregnancy [19,20]. Nevertheless, there is limited information globally and nationally in Vietnam about the number and timing of antenatal HIV test provided in sites where PMTCT services are available.

In Vietnam, the number of HIV-infections was 290,000 at the time of this study in 2007 and the HIV prevalence among adults 15-49 years was 0.5% [6]. The HIV prevalence among pregnant women was around 0.4% in 2008 [21]. However, the reported number of HIV-infected pregnant women may represent as little as a fourth of the real number [22]. Vietnam is one of the countries in Asia that adopted PMTCT at an early stage of the HIV epidemic and in the late 1990's a protocol for PMTCT prophylaxis was agreed. A comprehensive PMTCT programme, supported by Leadership and Investment in Fighting an Epidemic-Global AIDS Program (LIFE-GAP), has been piloted in five high HIV-prevalence provinces in 2004 and has become one of nine core programmes for the National Strategy on HIV/AIDS Prevention [23]. PMTCT services have since been integrated with antenatal and obstetric services at health facilities (HFs) at different levels. According to the programme, all pregnant women will be provided pre-test counselling and offered HIV testing when they have ANC. If women are detected to be HIV-positive, they will receive post-test counselling, antiretroviral (ARV) prophylaxis from 28 weeks of gestation until labour, and post-delivery care for both mother and infant. Therefore, women should be detected as HIV-positive before 28 weeks of gestation to receive the full regime of ARV prophylaxis and other care from PMTCT services. There is sporadic and limited evidence suggesting that a number of women are being tested multiple times during pregnancy. Hence concern has been raised that women who were initially tested at primary level will be tested again when they present at secondary or tertiary level due to lack of information sharing. Such repeat testing imposes unnecessary economic burdens on the individual women as well as on the health system. Particularly in resource constrained areas, such unnecessary expenses need to be acknowledged and addressed if possible. Concern about the timing of the HIV tests for pregnant women has also been raised. For example, it has been documented that 50-75% of HIV infected women are first diagnosed at the time of labour [4,24]. Based on this rationale, this study was conducted at a site where PMTCT has been scaled up. It explores the implementation of PMTCT with regard to number and timing of antenatal HIV tests. More specifically, the study aims to document the uptake of HIV testing among women attending ANC and to describe the extent of repeat testing and the timing of HIV testing. Results of this study will contribute evidence for making appropriate and effective guidelines for the National PMTCT programme.

## Methods

### Study setting

The study was conducted in Quang Ninh, a northern province of Vietnam with high rate of HIV (1.13%) in the population. The prevalence of HIV infection among women there has been around 1.25% in recent years [25]. This province is one of the five pilot sites for the PMTCT programme of the LIFE-GAP described in the Background.

Ha Long city, the study site, comprises 20 communes, separated into 3 main areas: Bai Chay (remote rural); Hon Gai (urban) and the outskirts of Hon Gai (semi-urban). For ANC and HCT, all communes in Ha Long have commune health stations, which are considered as primary health care facilities. There are two additional secondary HFs (Bai Chay hospital and Center of Maternal and Child Health Care) and one tertiary heath facility (Quang Ninh Provincial General Hospital).

In the city, ANC is available at all levels of the health care system, but delivery is only done at the secondary and tertiary HFs. The PMTCT services supported by the LIFE-GAP are available at the primary and the tertiary HFs. If at the time of labour a woman cannot provide evidence of having been tested for HIV within the past three months, she will be asked to have an HIV test. If the test result is positive, she is referred to the tertiary HF for support and treatment [4].

### Study design and data collection

**Figure 1:**
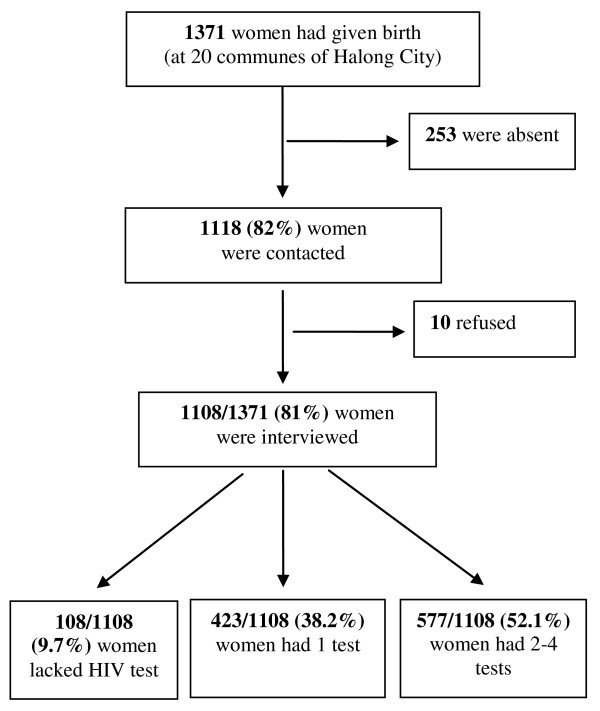
**Study population and HIV test during pregnancy**. This figure describes the process of selecting study population and the number of antenatal HIV test during pregnancy of the women.

A community-based cross-sectional study design was employed. Data were collected from April to September in 2007 among mothers who had given birth within 6 months from 1^st ^January - 30^th ^June 2007. The participants were recruited in all 20 communes of Ha Long, through the system of birth registration, which records all births at the community level. The names and addresses of the mothers and date of birth of their children were found in the records. In all, 1118 of the 1371 eligible women (82%) could be contacted and nearly all of them agreed to participate in the study. The 253 women were not present when the interviewers visited because they were either at work after maternity time (4 months after birth) or were staying at their mother's houses after the delivery. Of 1118 women who were invited to participate in the study, 10 refused to be interviewed. The total sample was thus 1108 women (Figure 1).

Face-to-face interviews were conducted following a structured questionnaire focusing on socio-economic characteristics of the households and the women's reproductive health history, including experience of ANC and of HIV testing during the last pregnancy.

The women who were not tested during pregnancy and those who did not know whether they were tested were grouped together and described as lacking an HIV test. The rationale for this grouping was that women who were not aware of being tested were in the same position as women who were not tested - both groups would not be able to take advantage of the PMTCT services offered, should they need them. The women who were tested more than once were described as having multiple tests, regardless of the total number of tests.

Data were entered into EPIDATA software and analyzed using SPSS for Windows, version 15.0. The variables under study were categorical; therefore, logistic regression analyses were used to assess the association between variables. The variables found to be significant at a p < 0.05 level were included in a multivariate regression analyses to calculate adjusted odd ratios. In this study, crude and adjusted odds ratios (ORs) were calculated with 95% confidence interval.

Written informed consent was obtained; the study was subject to guiding criticism from the Central Committee for Biomedical Research in Denmark. Ethical clearance was obtained from the Scientific Committee of General Office of Population and Family Planning, Ministry of Health, Vietnam. The authorities of Quang Ninh province and Ha Long city also approved the implementation of the study.

## Results

### Socio-economic characteristics of the women and number of HIV tests

The socio-economic characteristics of the women are presented in Table 1 together with the number of tests. Of the 1108 (100%) women, 672 (60.6%) were aged 25 to 34 and half were pregnant with their first child. All women had received schooling and the vast majority (94.7%) had secondary or higher educational level. Regarding their work, 431 (38.9%) were unemployed or housewives, 89 (8.0%) were farmers or seasonal workers while the others worked as Government staff, workers or had businesses. In all 300 (27.1%) were living in urban area, 395 (35.6%) in semi-urban area and 413 (37%) in remote rural area (Bai Chay).

**Table 1:** Socio-economic characteristics of interviewed women and number of antenatal HIV tests

Socio-economic characteristics of the women	Number of HIV test n (%)			Total	p-value
			
	Untested	1 test	2+ tests		
	*108 (9.7)*	*423 (38.2)*	*577 (52.1)*	*1108 (100)*	
**Age**					**0.405**
15-24	30 (27.8)	101 (23.9)	169 (29.3)	300 (27.1)	
25-34	63 (58.3)	269 (63.6)	340 (58.9)	672 (60.6)	
35-49	15 (13.9)	53 (12.5)	68 (11.8)	136 (12.3)	
**Number of children**					**0.002**
1	38 (35.2)	205 (48.5)	320 (55.5)	563 (50.8)	
2	67 (62.0)	204 (48.2)	241(41.8)	512 (46.2)	
3 - 4	3 (2.8)	14 (3.3)	16 (2.8)	33 (3.0)	
**Education level**					**0.024**
Primary school	10 (9.3)	25 (5.9)	24 (4.2)	59 (5.3)	
Secondary school	44 (40.7)	116 (27.4)	172 (29.8)	332 (30.0)	
High school	25 (23.1)	131 (31.0)	188 (32.6)	344 (31.0)	
College/University	29 (26.9)	151 (35.7)	193 (33.4)	373 (33.7)	
**Occupational**					**0.031**
Housewife/Unemployed	41 (38.0)	165 (39.0)	225 (39.0)	431(38.9)	
Farmer/Seasonal work	17 (15.7)	23 (5.4)	49 (8.5)	89 (8.0)	
Government staff/workers	27 (25.0)	142 (33.6)	183 (31.7)	352 (31.8)	
Business/other jobs	23 (21.3)	93 (22.0)	120 (20.8)	236 (21.3)	
**Residence**					**<0.0001**
Urban area	9 (8.3)	142 (33.6)	149 (25.8)	300 (27.1)	
Semi-urban area	17 (15.7)	128 (30.3)	250 (43.3)	395 (35.6)	
Remote rural area	82 (75.9)	153 (36.2)	178 (30.8)	413 (37.3)	
**Monthly income (million VND)**					**0.288**
<1.5	33 (30.6)	96 (22.7)	126 (21.8)	255 (23.0)	
1.5 - 2.5	27 (25.0)	101 (23.9)	129 (22.4)	257 (23.2)	
>2.5 - 3.5	28 (25.9)	133 (31.4)	172 (29.8)	333 (30.1)	
>3.5	20 (18.5)	93 (22.0)	150 (26.0)	263 (23.7)	

Regarding the number of antenatal HIV tests, most of the women (1000 of the 1108 or 90.3%) had been tested for HIV during pregnancy; 423 (38,2%) had been tested once and 577 (52,1%) had been tested twice or more. Among those in the untested group, 47 (4.3%) stated they had not been tested while 63 (5.5%) stated they did not know whether they had been tested or not.

### Factors associated with lack of antenatal HIV testing

Table 2 shows that the following socio-economic characteristics were associated with lack of antenatal HIV test: being pregnant with the second child (aOR 2.1, CI 1.3-3.4), being farmers or seasonal workers (aOR 2.2, CI 0.9-5.1), and living in a remote rural area (aOR 7.8, CI 3.4-17.8).

**Table 2:** Association between lack of antenatal HIV test and women's socio-economic characteristics, place and timing of first antenatal care and PMTCT information

Variables	Lack of test n (%)	Tested n (%)	p-value	Lack of test vs. Tested	
				
				Crude OR	Adjusted* OR
	***108 (9.7)***	***1000 (90.3)***			
**Age**			p = 0.8		
15-24	30 (10.1)	270 (90.0)		1.1 (0.7 - 1.7)	-
25-34	63 (9.4)	609 (90.6)		1.0	-
35-49	15 (11.0)	121 (89.0)		1.2 (0.7 - 2.2)	-
**Number of children**			p = 0.02		
1	38 (6.7)	525 (93.3)		1.0	1.0
2	67 (13.1)	445 (86.9)		2.1 (1.4 - 3.2)	2.1 (1.3 - 3.4)
3-4	3 (9.1)	30 (90.9)		1.4 (0.4 - 4.3)	0.6 (0.3-3.0)
**Education level**			p = 0.007		
Primary school	10 (16.9)	49 (83.1)		2.4 (1.1 - 5.3)	1.5 (0.5 - 4.5)
Secondary school	44 (13.3)	288 (86.7)		1.8 (1.1 - 3.0)	1.4 (0.7 - 2.7)
High school/vocational	25 (7.3)	319 (92.7)		0.9 (0.5 - 1.6)	0.7 (0.4 - 1.4)
College/University	29 (7.8)	344 (92.2)		1.0	1.0
**Occupational**			p = 0.01		
Housewife/Unemployed	41 (9.5)	390 (90.5)		1.3 (0.8 - 2.1)	1.2 (0.6 - 2.3)
Farmer/seasonal work	17 (19.1)	72 (80.9)		2.8 (1.5 - 5.5)	2.2 (0.9 - 5.1)
Government staff/workers	27 (7.7)	325 (92.3)		1.0	1.0
Business/Others jobs	23 (9.7)	213 (90.3)		1.3 (0.7 - 2.3)	1.4 (0.7 - 2.7)
**Residence**			p < 0.0001		
Urban area	9 (3.0)	291 (97.0)		1.0	1.0
Semi-urban area	17 (4.3)	378 (95.7)		1.5 (0.6 - 3.3)	1.9 (0.8 - 4.6)
Remote rural area	82 (19.9)	331 (80.1)		8.0 (4.0 - 16.2)	7.8 (3.4 - 17.8)
**Monthly income**(million VND)			p = 0.16		
<1.5	33 (12.9)	222 (87.1)		1.6 (1.0 - 2.8)	-
1.5 - 2.5	27(10.5)	230 (89.5)		1.3 (0.7 - 2.2)	-
>2.5 - 3.5	28 (8.4)	305 (91.6)		1.0	-
>3.5	20 (7.6)	243 (92.4)		0.9 (0.5 -1.6)	-
**HF for 1**^**st **^**ANC**			p < 0.0001		
Primary HFs	11 (6.1)	284 (95.9)		1.6 (0.7 - 3.6)	0.6 (0.2 - 1.7)
Secondary HFs	73 (13.7)	460 (86.3)		3.8 (2.0 - 7.0)	1.4 (0.7 - 3.0)
Tertiary HF	12 (4.1)	284 (31.1)		1.0	1.0
**Time of Gestation at 1**^**st **^**ANC**			p = 0.02		
< = 28 weeks	89 (9.1)	887 (90.9)		1.0	1.0
>28 weeks	7 (21.9)	25 (78.1)		2.8 (1.2 - 6.6)	3.6 (1.3 10.1)
					
**Provided Information about PMTCT at 1**^**st **^**ANC visit**			p < 0.0005		
No	92 (12.3)	657 (87.7)		9.0 (3.3-24.6)	7.4 (2.6 - 21.1)
Yes	4 (1.5)	256 (98.5)		1.0	1.0

* Adjusted for Number of children, Educational level, Occupation and Residence of the women.

With regard to the first ANC, women who accessed ANC later than at 28 gestational weeks (aOR 3.6, CI 1.3-10.1) were more likely to have missed having an HIV test than women who had attended ANC earlier. Women who attended HFs without PMTCT services (secondary HFs) were more likely to not be provided an antenatal HIV test (OR 3.8, CI 2.0-7.0), than women who attended the HFs where PMTCT services were available. However, the association was not significant after controlling for confounding factors (aOR 1.4, CI 0.7-3.0). Moreover, women who were not provided information on PMTCT during the first ANC visit were at higher risk of lack of HIV test (aOR 7.4, CI 2.6-21.1), in comparison with women who were informed about HIV testing.

### Factors related to the number of HIV testing during pregnancy

Table 3 shows the association between the number of tests and the women's socio-economic status, HF of first ANC visit, and first HIV test. There were almost no significant differences in socio-economic characteristics between women who were tested once and women tested several times. Among the tested women, the women who were living in a semi-urban area were more likely to have been tested twice or more.

**Table 3:** Association between number of tests and women's socio-economic characteristics and health facility of first antenatal care visit and first HIV test

Variables	1 test n (%)	2+ tests n (%)	p-value	2+ tests vs. 1 test	
				
				Crude OR	Adjusted* OR
	***423 (42.3)***	***577 (57.7)***			
**Age (years)**			p = 0.16		
15-24	101 (37.4)	169 (62.6)		1.3 (1.0 - 1.8)	
25-34	269 (44.2)	340 (55.8)		1.0	
35-49	53 (43.8)	68 (56.2)		1.0 (0.7 - 1.5)	
**Number of children**			p = 0.09		
1	205 (39.0)	320 (61.0)		1.3 (1.0 - 1.7)	
2	204 (45.8)	241(54.2)		1.0	
3-4	14 (46.7)	16 (53.3)		1.0 (0.5 - 2.3)	
**Education level**			p = 0.46		
Primary school	25 (51.0)	24 (49.0)		0.7 (0.4 - 1.4)	
Secondary school	116 (40.3)	172 (59.7)		1.2 (0.9 - 1.6)	
High school/vocational	131 (41.0)	188 (58.9)		1.1 (0.8 - 1.5)	
College/University	151 (43.9)	193 (56.1)		1.0	
**Occupational**			p = 0.31		
Housewife/Unemployed	165 (42.3)	225 (57.7)		1.1 (0.8 - 1.4)	
Farmer/seasonal work	23 (31.9)	49 (68.1)		1.7 (1.0 - 2.8)	
Government staff/workers	142 (43.7)	183 (56.3)		1.0	
Business/Other jobs	93 (43.7)	120 (56.3)		1.0 (0.7 - 1.4)	
**Residence**			p < 0.0001		
Urban area	142 (48.8)	149 (51.2)		1.0	1.0
Semi-urban area	128 (33.9)	250 (66.1)		1.9 (1.4 - 2.6)	1.8 (1.3 - 2.6)
Remote rural area	153 (46.2)	178 (53.8)		1.1 (0.8 - 1.5)	0.5 (0.3 - 0.7)
**Monthly income **(million VND)			p = 0.54		
<1.5	96 (43.2)	128 (56.8)		1.0 (0.7 - 1.4)	
1.5 - 2.5	101(43.9)	129 (56.1)		1.0 (0.7 - 1.4)	
2.5 - 3.5	133 (43.6)	172 (56.4)		1.0	
>3.5	93 (38.3)	150 (61.7)		1.3 (0.9 - 1.8)	
**HF of 1**^**st **^**HIV testing**			p < 0.0001		
Primary HFs	114 (26.3)	320 (73.7)		4.7 (3.5 - 6.4)	6.8 (4.4 - 10.6)
Secondary HFs	97 (42.7)	130 (57.3)		2.2 (1.6 - 3.2)	4.9 (2.9 - 8.1)
Tertiary HF	212 (62.5)	127 (37.5))		1.0	1.0
**HF of 1**^**st **^**ANC visit**	381 (41.8)	513 (58.2)	p < 0.0001		
Primary HFs	46 (27.4)	122 (72.6)		2.9 (1.9 - 4.3)	1.0 (0.6 - 1.7)
Secondary HFs	188 (40.9)	272 (59.1)		1.6 (1.2 - 2.1)	0.8 (0.6 - 1.3)
Tertiary HF	147 (51.8)	137 (48.2)		1.0	1.0

* Adjusted for Residence of the women.

With regard to the HF, the women who had had their first HIV test and their first ANC visit and at primary level were more likely to have been tested twice or more (OR 4.7, CI 3.5-6.4 and OR 2.9, CI 1.9-4.3). In the multivariate analysis, the association between HFs of the first HIV test became stronger; women who were tested at primary HFswere found to be 7 times more likely to have been tested twice or more (aOR 6.8 CI 4.4-10.6). The association between HFs at first ANC visit and the number of tests became insignificant after controlling for confounding factors, whereas the association between place of residence and number of tests remained the same.

The data presented in Table 4 provide additional information about women who were tested more than once, showing the gestational period when the second HIV test was done. Only 20.1% of the women who were tested multiple times had their second test before 36 gestational weeks, while 76.8% were tested for the second time at labour.

**Table 4:** Gestational stage at second HIV testing

**Gestational stage at 2**^**nd **^**test**	Frequency	Percentage
	**577**	**100**
<36 wks	116	20.1
> = 36 wks	18	3.1
At the time of labour	443	76.8

## Discussion

The results of this study showed that 10% of the women in the study sample were never, to their knowledge, tested for HIV during their pregnancy. Among those who were tested, more than half (577/1000) were tested more than once and 82% (472/577) had their second test after 36 gestational weeks.

Lack of HIV testing was uncommon in the present study, where only 10% of the women were either not tested or were not aware of having been tested for HIV during their most recent pregnancy. Missing information about HIV testing during the first ANC visit was the main factor associated with lack of HIV testing. This finding is in line with other studies, for instance, studies from Uganda and India reporting that only 10% and 3%, respectively, of pregnant women had had antenatal HIV tests [5,13]. These studies further documented that a lack of counselling on HIV testing during ANC visits and lack of awareness about availability of HCT services were the main reasons why the women were not tested. In addition to missing information about PMTCT, important factors for low uptake of HIV testing were: having poor education, low income, working as a farmer, living in a semi-rural area and going late for ANC. The association between poor socioeconomic status and poor uptake of PMTCT has likewise been documented in a study from the neighboring Hai Phong province [24] as well in other studies from Asia [5,9]. Further, recent studies from Hanoi revealed that poor antenatal counselling and lack of HCT services at community level, as well as being offered HIV testing at a late stage of pregnancy resulted in women not being tested for HIV during ANC [14,26].

Regarding the number of tests, more than half of the women had been tested more than once during pregnancy. According to the recommendation on repeated antenatal HIV testing, the CDC lists two criteria which should be considered when implementing repeat HIV testing: Firstly, the prevalence rate of HIV among pregnant women should be 0.1% or above in the environment of the health care setting; secondly, the women should be at high risk of HIV infection and/or they should have symptoms consistent with acute HIV infection [12:9]. In the Vietnamese context, the benefits of providing multiple HIV testing during pregnancy may be discussed. Firstly, the HIV prevalence rate among pregnant women is generally low, although it varies greatly by province; the rates are equal to or less than 0.1% in 45 provinces and 0.2 - 1.4% in 12 provinces [27]. Supporting this point, reports from Indonesia and India have demonstrated the accuracy of the one-time rapid test, indicating that the positive predictive value of the first screening HIV test was 100% and the risk of a false-positive rapid test was low among the general population and among pregnant women [28,29]. Furthermore, recent figures from UNAIDS suggest that only 2% of Vietnamese women had sexual intercourse with more than one partner during one year [6]. The figure may be even lower if only married women are considered. This low-risk sexual behavior is also reflected in a number of studies documenting very low rates of sexual transmitted infections, ranging from 2-10% [30,31]. Against this background it may be argued that the risk of sero-conversion during pregnancy is low in Vietnam and the benefit of repeated HIV testing may thus be questioned. Especially considering that the women have to pay approximately 3 US dollars (50.000 VND) for an HIV test, which is substantial amount of money in the Vietnamese context where 50% of the population have a daily income below 2 USD [32] and 24% of the population live below the International Poverty Line of 115 USD (1.92 million VND/) per year [33]. In addition to the direct cost to the women, the indirect costs such as time spent by both women and health staff as well as the pressure added to an already constrained health system should be considered. Moreover, financial support for PMTCT programmes in Vietnam has mainly come from foreign donor agencies [34] and concern prevails about this aid being reduced in the near future due to the global financial crisis and the economic development in Vietnam [35]. Within this perspective, it may be argued that unnecessary repeated HIV testing may add burdens in an already resource constrained country where medical services need to be prioritized. Hence, as suggested in a recent study focusing on the scale up of HIV care and treatment, services among uninfected individuals should be prioritized to reduce the financial burden for the users and for the health care system [20]. Moreover, the results of this study showed that women who were tested at primary level HFs had a higher risk of having the repeat tests when they presented at higher level HFs. This happened partly because of the lack of integration and of communicating HIV test results between HFs [26].

Focusing on timing of the second antenatal HIV test, it is recommended that pregnant women should be offered a second HIV test *before *the 36^th ^week of gestation [1,11]. However, we found that 80% of the women had had the second test *after *36 gestational weeks. Such late testing will reduce the effect of the PMTCT service offered. Among the women who had the second test after 36 weeks, the vast majority were tested at the time of labour. The high rate of HIV tests performed at labour may reflect health staff's concerns about occupational exposure to HIV, their concern about risk of transmitting HIV from one delivering woman to another as well as efforts made to detect new HIV infections for case management [4]. Results of the study show that the women who had the first HIV test at primary level HFs were more likely also to be tested at labour. This finding may be explained by the weak communication of HIV test results between HFs [4,14]. With regard to the benefits of HIV testing, although HCT around the time of labour might be considered as a way to increase PMTCT uptake and as the last opportunity for prevention of MTCT [1,19,29], the detection of HIV only at the time of labour reduces the benefit for women, and their infants are still at high risk of HIV-infection [4,35]. Additionally, communication of a positive test result at the time of labour may result in even greater distress for both women and health staff [36,37]. Such a situation could be avoided if the health staff were to discuss the timing of the second HIV test in relation to a woman's first antenatal HIV test and decide on the necessity of performing it.

The results of this study may have been affected by selection bias. We planned to include all 1371 women who had given birth within the last half year, but 253 (19%) were absent when we sought to interview them. Since we did not collect background information on these women we cannot reject the possibility that they differed systematically in terms of socio-economic characteristics and health-seeking behavior from the women who were included in the study. If they were less likely to attend ANC and to be tested for HIV, their absence could have had an effect on the results. On the other hand, 81% of the eligible women did participate in the study and it may be argued that due to this high participation rate, the risk of selection bias is not great. Information recall bias might also be an issue in the present study, where the women were interviewed 1- 6 months after they had given birth. It might have been difficult for the individual women to recall exactly the information related to ANC or the timing of each HIV test. We tried to control for this problem by designing different questions for the same answer and cross-checking the answers obtained.

Massive investments have been made in PMTCT in Quang Ninh province, which has served as a pilot PMTCT site, therefore our findings can not be generalised to other parts of Vietnam where less money and resources have been invested in PMTCT. However, the findings from this pilot area may provide useful information as PMTCT is further scaled up in those other areas of Vietnam, as well as in other low income countries.

## Conclusions

The results of this study showed that a lack of HIV test during pregnancy was associated with poor socio-economic status and with lack of information and offering HCT services at the first ANC visit.

We found that many women had been tested more than once. The provision of a repeat test should be reviewed in the context of Vietnam, a resource-limited setting where prevalence of HIV among pregnant women is near the limit of 0.1% and where women usually have stable sexual lives. Another issue is that many women in this study were tested twice or more and many were tested at the time of labour. To ensure that women will have repeat tests in an appropriate and timely way, health staff should provide adequate post-test counselling for HIV-negative pregnant women, and discuss about timing for a repeat test (if necessary) when they have the results of the first HIV test.

## Competing interests

The authors declare that they have no competing interests.

## Authors' contributions

NTTH participated in designing the study and revised the protocol, conducted the data collection and performed the statistical analysis; outlined, drafted and revised the manuscript. TG participated in designing the study and revised the protocol, provided critical comments for finalizing the paper. VR participated in designing the study and revised the protocol, outlining the manuscript, and provided critical comments through the writing process. All authors read and approved the final manuscript.

## Pre-publication history

The pre-publication history for this paper can be accessed here:

http://www.biomedcentral.com/1471-2458/11/183/prepub
